# S110. THE CLINICAL IMPLICATION OF CLINICIAN-RATED DIMENSIONS OF PSYCHOSIS SYMPTOM SEVERITY (CRDPSS) FOR DIAGNOSIS BY DSM-5

**DOI:** 10.1093/schbul/sby018.897

**Published:** 2018-04-01

**Authors:** Beomwoo Nam, Won-Myong Bahk, Sang-Yeol Lee, Kwanghun Lee, Duk-In Jon, Eunsung Lim, Sung-Yong Park, Min-Kyu Song, Seongwoo Jo, Youngsoon Jeon

**Affiliations:** 1 Konkuk University; 2 Yeouido St. Mary’s Hospital, The Catholic University of Korea; 3 Wonkwang University, School of Medicine; 4 College of Medicine, Dongguk University; 5 College of Medicine, Hallym University; 6 Shinsegae Hospital; 7 Keyo Hospital; 8 Yesan Mental Health Clinic

## Abstract

**Background:**

The most recently published the 5th edition of the DSM proposed a dimensional approach with continuous of schizophrenia and other psychoses. The newly proposed Clinician-Rated Dimensions of Psychosis Symptom Severity (CRDPSS) in the DSM was recommended to be evaluated in all disorders with psychotic symptoms in eight dimensions; Hallucinations, Delusions, Disorganized speech, Abnormal psychomotor behavior, Negative symptoms, Impaired cognition, Depression, Mania. The purpose of this study is to examine if Clinician-Rated Dimensions of Psychosis Symptom Severity (CRDPSS) can usefully be used for the Non-Affective Psychoses (NP) and Affective Psychoses (AP).

**Methods:**

Participants in the study were 175 diagnosed with Schizophrenia, or Schizophreniform Disorder, Schizoaffective Disorder, mood disorder with psychotic symptoms (Major Depressive Disorder, Bipolar Disorder) based on DSM-5 diagnostic criteria and were assigned to either the NP (n = 154) or AP (n = 21) group. CRDPSS was performed jointly by a psychiatrist and a psychiatric resident to assess the severity of the psychotic symptoms of all the participants. And WHO Disability Assessment Schedule 2.0 (WHODAS 2.0) was responded to all participants. Independent T-test was conducted to determine whether there was a difference in CRDPSS profile and WHODAS 2.0 scores between the two groups. In addition, a linear discriminant analysis was performed to determine whether the CRDPSS profile can discriminate between the two groups.

**Results:**

Demographics and WHODAS 2.0 had no statistically significant differences between the two groups. On the other hand, Patients in the NP group had higher Hallucination (p < .05) and Negative symptoms (p < .001), however, lower Mania (p < .001). As a result of constructing a linear discriminant function for NP and AP, the correct classification rate of CRDPSS to discriminate between two groups was 84%.

**Discussion:**

The results of this study are the first to distinct effectively that Non-Affective psychoses and Affective psychoses by CRDPSS profile. There was no difference in the level of functional disability between groups NP and AP, but only CRDPSS profile could discriminate both groups. Hallucinations, Negative symptoms, and Mania were the major contributors to the distinction between the two groups. This is consistent with the previous studies that these are important in distinguishing Schizophrenia and Bipolar Disorder from each other. CRDPSS provides a new perspective that can be viewed from an integrated perspective, the NP and AP. Regarding the result of this study that it is more important to identify the score profile than the combined score of CRDPSS, because patients exhibit very heterogeneous profile of symptoms.

